# Protic Stabilization Engenders High Energy Density and Long Cycle Life in Polyaniline–Zinc Supercapacitors

**DOI:** 10.1002/smsc.202400295

**Published:** 2024-09-01

**Authors:** Chanho Shin, Eun Hye Lee, Hyeong Ju Eun, Jinwook Jung, Jong H. Kim, Tse Nga Ng

**Affiliations:** ^1^ Program in Material Science and Engineering University of California San Diego 9500 Gilman Drive La Jolla CA 92093 USA; ^2^ Department of Electrical and Computer Engineering University of California San Diego 9500 Gilman Drive La Jolla CA 92093 USA; ^3^ Department of Molecular Science and Technology Ajou University Suwon 16499 Republic of Korea

**Keywords:** polyaniline, supercapacitors, zinc ion capacitors

## Abstract

The redox activities of polyaniline (PANI) are hindered by the instability of pernigraniline salt (PS) state which degrades into oligo‐aniline. In this work, the use of protic additives is examined to mitigate capacity fading and increase utilization of PANI in nonaqueous electrolytes. The protic additive propylene glycol, with its hydrogen‐bonding capabilities, stabilizes the PS PANI and promotes reversible redox reactions, facilitating high capacity and an extended cycle lifetime for applications in metal ion supercapacitors. The use of this protic nonaqueous electrolyte in a PANI–zinc device results in an energy density of 255 Wh kg^−1^ at a power density of 1.8 kW kg^−1^ and a robust cycle lifetime of 3,850 charge/discharge cycles. The PANI at a high current density of 6.5 mA cm^−2^ reaches a capacity of 257 mAh g^−1^, equivalent to 87% of the its theoretical capacity, showcasing the effectiveness of the protic additive in improving both capacity and cycle life in electrochemical supercapacitors.

## Graphical abstract

The redox activities of polyaniline (PANI) are hindered by the instability of pernigraniline salt state which degrades into oligo‐aniline. In this work, the use of protic additives is examined to mitigate capacity fading and increase utilization of PANI in nonaqueous electrolytes, facilitating high capacity and an extended cycle lifetime in zinc‐ion supercapacitors.

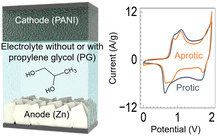

## 1 Introduction

Redox‐active polymers offer the potential to improve electrode materials for electrochemical energy‐storage systems.^[^ ^1^, ^2^, ^3^, ^4^, ^5^ ^]^ Compared to conventional transition metal oxides, polymers enable faster kinetics and exhibit greater tolerance to the mechanical deformation from ion movement. One of the most frequently used redox‐active polymers is polyaniline (PANI), which is low cost and scalable and has a high theoretical capacity of 294 mAh g^−1^.^[^ ^6^ ^]^ However, despite its long history, PANI cathodes have shown limited redox reversibility,^[^ ^7^ ^]^ restricting their practical capacity and cycle life particularly in nonaqueous conditions. Opportunely, recent mechanistic studies^[^ ^6^, ^8^ ^]^ have revealed critical degradation pathways related to the degree of protonation in PANI, inspiring this work to examine the role of hydrogen bonding using protic additives to overcome capacity fading and increase utilization of PANI in nonaqueous electrolytes.

PANI has been used in both aqueous and nonaqueous energy‐storage cells. While PANI is stable as emeraldine salt (ES) and nigraniline states,^[^ ^9^, ^10^ ^]^ its operational voltage window is constrained by unstable pernigraniline salt (PS), hindering PANI from undergoing redox across the full potential range to maximize energy and power density. If PANI can be oxidized at high potentials (up to 2 V versus Ag/Ag^+^ reference) its charge storage capacity would markedly increase. However, achieving redox reversibility becomes challenging in PS PANI, resulting in an irreversible pathway from PS to oligo‐anilines.^[^ ^6^, ^11^ ^]^ The irreversibility arises from the detachment of oligo‐anilines which are formed by side reactions between PANI and the electrolyte solvent.^[^ ^8^ ^]^ Therefore, although the formation of oligo‐aniline temporarily increases the capacity because of the redox activities of its functional groups, the eventual detachment of oligo‐anilines from the cathode decreases its capacity after a few cycles.

**Figure 1:**
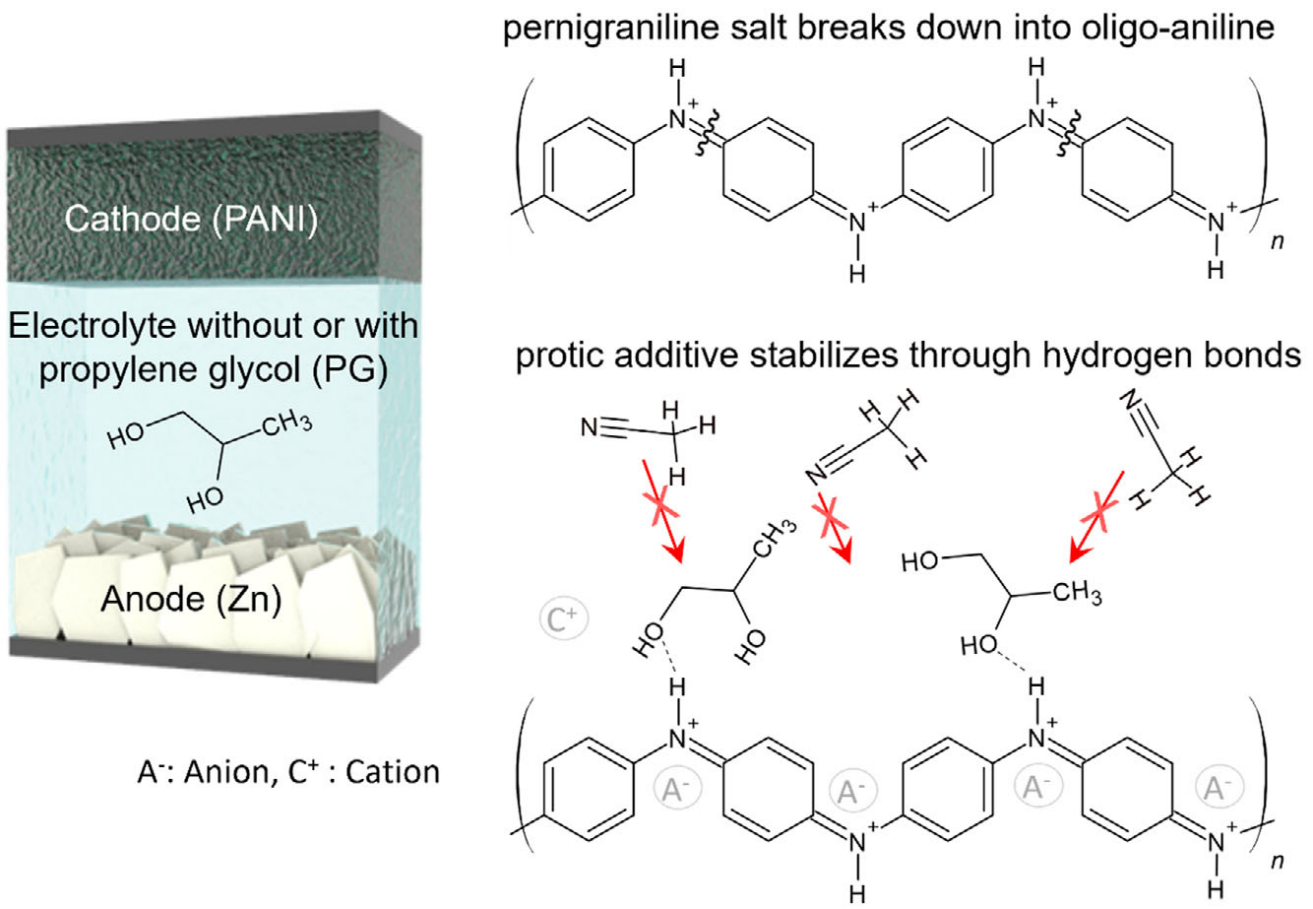
Influence of the protic additive on polyaniline–zinc supercapacitors. The presence of hydrogen bonding reduces the contact between the acetonitrile solvent and polyaniline to minimize the degradation of PS into oligomers.

Considering this reversibility issue, fully oxidized PANI can be in either the oligo‐aniline or the PS form as shown in **Figure** **1**. Instead of following the irreversible pathway to the oligo‐aniline, stabilized PS form would potentially promote reversibility, since intact polymer chains can favor stability due to π–π interchain interaction. Yet there is difficulty with stabilizing PS, which is highly susceptible to nucleophilic attack by electrolyte. Prior works^[^ ^11^, ^12^ ^]^ incorporated a polyacid copolymer that provided hydrogen bonds around PANI to stabilize the PS state, demonstrating 88.5% capacity retention after 800 cycles in a nonaqueous electrolyte. While this copolymer approach extended the redox cycle life of PANI, the polyacid added substantial mass and lowered the copolymer's gravimetric capacity by half compared to the homopolymer. Therefore, in this study, we investigate a different PS stabilization strategy for the homopolymer PANI, to increase its capacity and cycle life over a wide voltage window in nonaqueous electrolytes.

To stabilize the fully oxidized PS PANI, we hypothesize that adding a small quantity of protic solvent^[^ ^13^ ^]^ into nonaqueous electrolytes would facilitate hydrogen bonding, thereby stabilizing the PS state while avoiding hydrolysis and minimizing the formation of oligo‐aniline. The protic additive used here is propylene glycol (PG), selected for its structure with two hydroxyl (—OH) groups per molecule to increase the density of the hydrogen bond and minimize side reactions. The molecular structure of PANI is monitored by in situ Raman spectroscopy, enabling a comparison of how the protic additives influence the redox states and stability of the polymer.

Building upon the protic additive strategy, we proceed to develop an electrochemical supercapacitor comprising a PANI composite cathode and a zinc (Zn) anode. Rechargeable Zn‐ion cells^[^ ^14^, ^15^ ^]^ are appealing because of their advantages in safety and Zn being an earth‐abundant element. In previous Zn‐ion capacitors, carbon materials were predominantly employed in the cathode to attain good cycling stability, constrained by a trade‐off between capacity and cycle life. Here, we combine PANI and reduced graphene oxide (rGO)^[^ ^8^, ^16^ ^]^ to increase the cathode capacity while enhancing the cycling stability of the supercapacitor through the protic additive. This work culminates in demonstrating state‐of‐the‐art energy‐storage performance, cycle life, and self‐discharge characteristics of the protic PANI–Zn system.

## 2 Results and Discussion

### 2.1 Structural Changes of PANI in Aprotic Versus Protic Electrolyte

**Figure 2:**
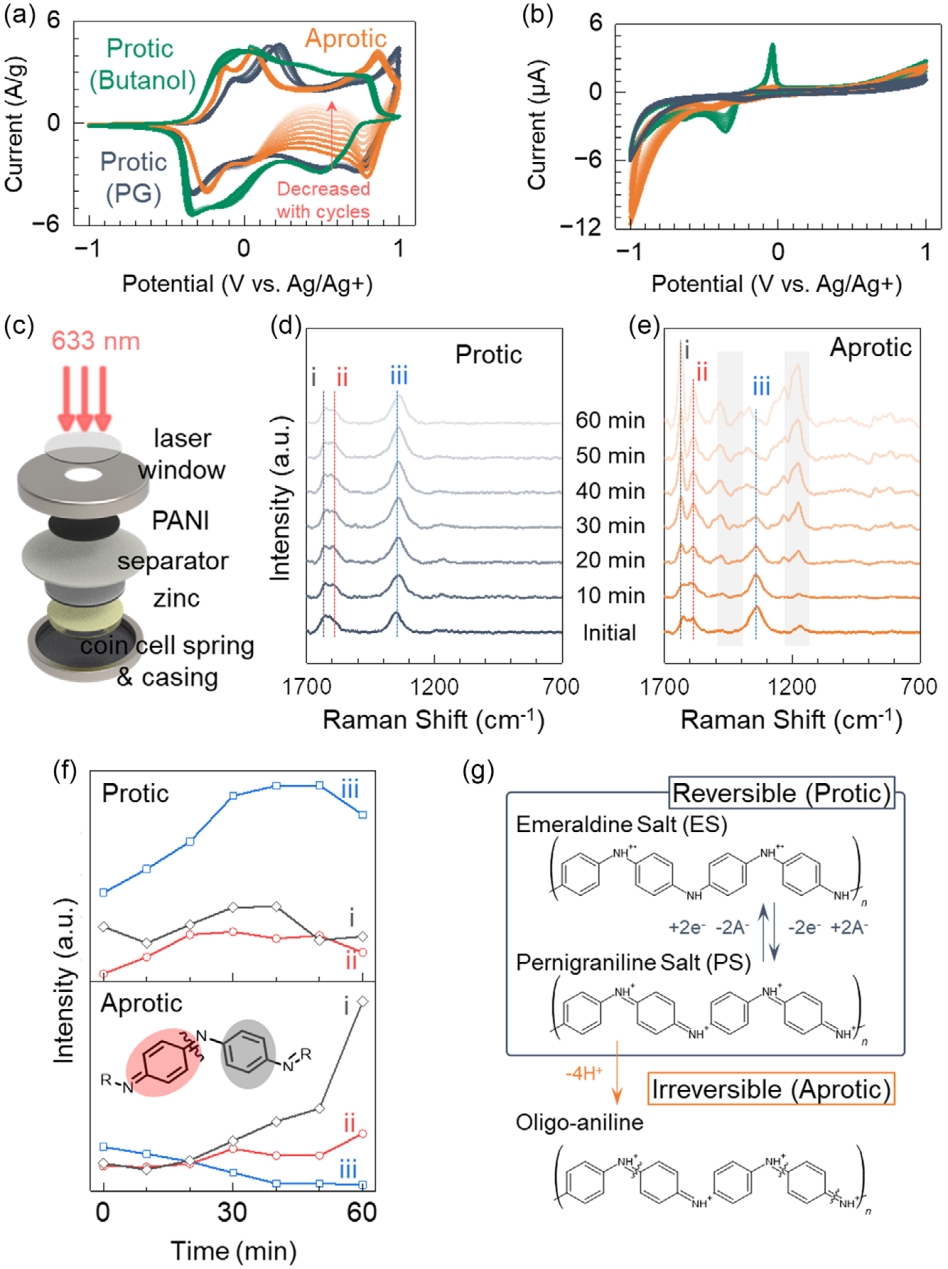
Current–voltage characteristics with aprotic and protic electrolyte versus Ag/Ag^+^ reference; a) PANI cathode and Zn anode, and b) glassy carbon electrodes. Comparison of PANI structures undergoing redox in the protic or aprotic electrolyte. c) Device structure used for in situ Raman spectroscopy. Raman spectra of PANI as a function of time under a constant bias of 2 V on a cell d) with or e) without the protic PG additive at 5% volume. f) Intensity changes of the Raman peaks attributed to the moieties of benzenoid (i: 1,630 cm^−1^), quinoid (ii: 1,590 cm^−1^), and polaron (iii: 1,340 cm^−1^), extracted from parts (d,e), respectively. The inset illustrates the benzenoid and quinoid segments and a potential bond cleavage site. g) The schematics show the different forms (ES, PS, and oligo‐aniline) of PANI.

To investigate the effect of protic additives, compounds with similar molecular structure such as 2‐butanol (BuOH), ethanolamine (ETA), and PG were added at 5% by volume to the aprotic electrolyte solvent, which was acetonitrile (ACN) in this study. **Figure** **2**a shows the cyclic voltammetry characteristics of protic and aprotic electrolytes in 3‐electrodes configuration. While the electrode in the aprotic electrolyte showed an initially high oxidation current due to side reactions that led to oligo‐aniline, the reduction current of the fragmented PS was much lower and continued to decrease with successive redox cycles as shown in Figure 2a. Meanwhile, the oxidation and reduction currents in the protic electrolytes were balanced, indicating no loss of redox activities in switching between the protonated ES and PS states. The protic additives provided hydrogen bonds screening the PANI from ACN, to lower the likelihood of side reactions and enhance reversibility.

Among the protic additives we tested, BuOH and ETA showed unstable current–voltage characteristics due to their narrow potential window and redox activities between −0.05 and −0.35 V versus the Ag/Ag^+^ reference, as seen in Figure 2b and S1, Supporting Information, taken with glassy carbon working electrodes. Additionally, the protic electrolyte with ETA (pKa 9.5) inhibited the second redox peak in PANI in Figure S1, Supporting Information. This observation indicated that an additive with a strong basic characteristic hindered the oxidation of PANI. Meanwhile, the PG additive was not reactive within the 2 V potential window in Figure 2b. PG was considered a weak base, and adding a few percent of PG into a solvent would not induce a strongly basic environment. Hence, PG was used as the protic additive for the rest of this study.

The molecular structure of the PANI cathode was monitored by in situ Raman spectroscopy as depicted in Figure 2c. The optical window on the coin cell allowed the transmission of the laser excitation beam and the reflected light signal subsequently collected by the spectrometer. Figure 2d,e shows the temporal evolution of the Raman spectra when the PANI cathode was under a constant bias of 2 V in a cell with or without the protic PG additive, in the nonaqueous electrolyte of 0.5 m zinc triflate in ACN. The peaks^[^ ^17^, ^18^, ^19^ ^]^ attributed to C—C stretches in a benzenoid ring (1,630 cm^−1^), C=C stretches in a quinoid ring (1,590 cm^−1^), and C∼N^+^ stretches in a polaron (1,340 cm^−1^) are denoted by the 1) black‐, 2) red‐, and 3) blue‐dashed lines, respectively.

The intensity changes of the denoted peaks were compared overtime in Figure 2f, to infer the PANI structure resulting from oxidation in either the protic or aprotic electrolyte. In the protic electrolyte, intensities of the benzenoid and quinoid peaks stayed relatively constant overtime while the polaron peak increased, which was indicative of a stable coexistence state of ES and PS forms. Also, PG helped to keep PANI in stable PS form and not degraded into the oligo‐aniline, improving the reversibility of protonation and deprotonation at a relatively low concentration of the additive.

In contrast, the opposite trend was observed in the aprotic electrolyte; the benzenoid and quinoid peaks increased whereas the polaron peak dropped in intensity. Moreover, additional signals arose in the shaded regions of Figure 2e, with the 1,160 cm^−1^ peak attributed to C—H bending of quinoid rings and the 1,480 cm^−1^ peak corresponding to C—N stretches in benzoquinone.^[^ ^19^ ^]^ The appearance of these new peaks, heightened intensities of benzenoid and quinoid stretches, and the decrease of polarons collectively suggested a degradation of the polymer into smaller oligo‐aniline fragments. These results corroborate electrochemical instability of PANI cathode in aprotic electrolyte. Thus, oxidation of PANI to the oligo‐aniline state in an aprotic environment was unfavorable for stability.

The ultraviolet–visible (UV–vis) light absorption characteristics and additional Raman spectra taken under different voltages in Figure S2, Supporting Information, show a decrease of quinoid intensity. The fragmentation of PS form corresponded to less quinoid and eventual detachment of oligo‐aniline from the cathode. By tracking the polaron peak overtime as presented in Figure 2f, the decrease in peak intensity was fitted to a first‐order rate equation *I *= *I*_0_ exp(‐*bt*) where *I* is the peak intensity at a given time, *I*_0_ is the initial intensity, *t* is the reaction time, and *b* is the first‐order reaction rate. The data fitted very well to the first‐order equation as shown in Figure S2, Supporting Information, suggesting that the degradation was dependent mainly on the concentration of oligo‐aniline and not a second‐order reaction with other molecules in the electrolyte. The first‐order rate was found to be *b* = 6.2 × 10^−4^ s^−1^. This value for oligo‐aniline state was comparable to the degradation rates reported for PANI in other studies conducted in aqueous electrolytes.^[^ ^18^ ^]^ In contrast, for the coexistence state of ES and PS PANI, the intensity of the polaron peak remained constant, and thus it was not applicable to extract a decay rate.

Thus, the spectroscopic data provided clarity on why the capacity faded away in aprotic electrolytes. The degraded oligo‐aniline was a main contributing factor to irreversibility. The cyclic voltammetry data in Figure 2a corroborated this finding, that while the electrode in the aprotic electrolyte showed an initially high oxidation current due to side reactions that led to oligo‐aniline, the reduction current of the fragmented PS was much lower and continued to decrease with successive redox cycles. Meanwhile, the oxidation and reduction currents in the protic electrolyte were balanced, indicating no loss of redox activities in switching between the protonated ES and PS states. The schematics in Figure 2g (and a more detailed version in Figure S3, Supporting Information) illustrate the reversible redox processes between ES and PS, as well as the irreversible oxidation to oligo‐aniline in aprotic conditions that impact electrode stability.

### 2.2 Optimization of the Protic Electrolyte and PANI–rGO Cathode to Improve Cycling Stability

**Figure 3:**
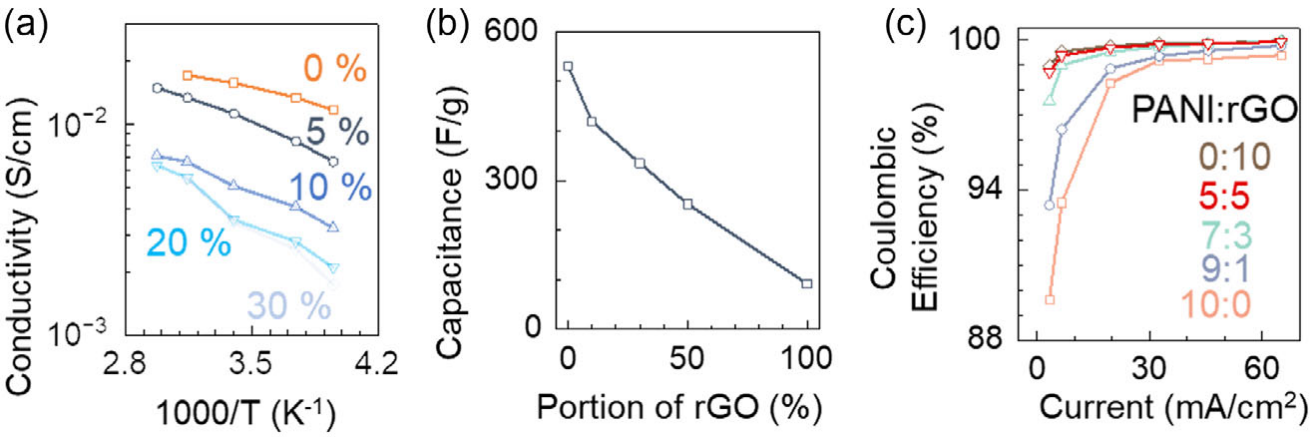
Optimization of the electrolyte and the cathode. a) Ionic conductivity of 0.5 m zinc triflate electrolyte with different percentages of propylene glycol additive in the temperature range from −20 to 63 °C. b) Specific capacitance versus PANI:rGO ratios in an electrolyte with 5% additive. c) Coulombic efficiency of the cathode, measured by galvanostatic charge–discharge cycles taken at current densities of 3.25–65 mA cm^−2^.

Given that the protic additive was shown to be effective at stabilizing the oxidized PANI cathode, various concentrations of the additive were examined to assess its effect on the ionic conductivity of the electrolyte. **Figure** **3**a displays the change in ionic conductivity as a function of temperature and volume ratios of PG added to the electrolyte. The ionic conductivity decreased as the concentration of the additive increased, likely due to the higher viscosity of PG (42.9 mPa s) in comparison to the ACN solvent (0.334 mPa s) and the change in ion solvation structure with the additive. The increased viscosity of the electrolyte mixture led to slower ion movement with higher concentrations of additives. Consequently, we chose the volume ratio of 5% additive for use in the full cells, to balance between the requirement for high ionic conductivity and sufficient proton‐donating capacity to maintain cathode stability.

Another optimization step involved creating a composite cathode by combining PANI with rGO. While the PS PANI was stabilized by hydrogen bonds with the protic additive, the electrode volume change^[^ ^20^, ^21^ ^]^ during redox reaction would impact electrical properties. Previously polymer–rGO composites^[^ ^8^, ^22^, ^23^ ^]^ have shown improvements in electrode conductivity and cycle life through the introduction of extended π–π interaction to enhance electrical connections and mitigate materials dissolution. However, because the specific capacitance of rGO (≈200 F g^−1^) is lower than that of PANI (≈700 F g^−1^),^[^ ^8^, ^24^ ^]^ increasing the ratio of rGO to PANI will lead to a decrease the electrode capacitance. Hence, in Figure 3b,c, we characterized various ratios of PANI:rGO to identify the composition that would optimize both specific capacitance and Coulombic efficiency (CE, i.e., the reversibility in storing and releasing charge). With increasing the ratio of rGO in the composite, the specific capacitance dropped, but concurrently the CE improved, especially in the low current density regime below 20 mA cm^−2^. Considering that the CE plateaued around the ratio of 7:3 PANI:rGO, there would be negligible benefit with adding more rGO beyond this point. Then, the cathode composition of 7:3 PANI:rGO was selected to be paired with an electroplated Zn anode for the supercapacitor fabrication.

### 2.3 PANI–rGO||Zn Supercapacitors with Aprotic versus Protic Electrolyte

In this report, the zinc‐ion supercapacitors used a capacity ratio between the negative and positive electrodes (n/p ratio) down to 2.06. While the n/p ratio should ideally be 1, prior works^[^ ^14^, ^25^ ^]^ often used high n/p ratio >5, i.e., excess zinc, since the cathodes were mainly carbon materials based on electric double layer (EDL) with low capacity. The unbalanced n/p ratio reduced the energy density of the devices. Here we lowered the n/p ratio of our devices and checked their operational mechanisms by the Dunn model^[^ ^26^ ^]^ (current versus voltage scan rates in Figure S4, Supporting Information). The contribution from the capacitive current was shown to be at least 71%, with the remaining contribution attributed to the redox current. Thus, the devices in this report were categorized as pseudo/super‐capacitors instead of redox‐dominant batteries. With this classification, we assessed the self‐discharge characteristics^[^ ^27^, ^28^ ^]^ and cumulative capacity for supercapacitors, along with typical power and energy metrics for electrochemical cells. The following characterization also compared the differences in device performance arising from the incorporation of the protic additive in the electrolyte.

**Figure 4:**
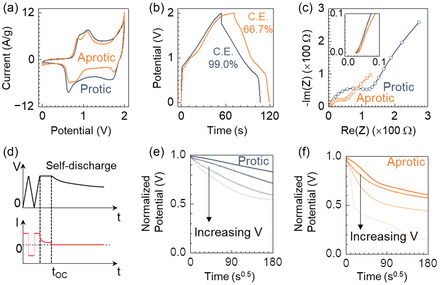
Performance comparison of PANI–rGO||Zn supercapacitors with (blue) and without (orange) propylene glycol. a) Cyclic voltammetry at a scan rate of 10 mV s^−1^. b) Galvanostatic charge–discharge measurements at a current density of 6.5 mA cm^−2^. c) Real versus imaginary impedance in the frequency range from 50 mHz to 200 kHz at 0 V. d) Schematics of the potential (black line) and current (red line) profiles in self‐discharge measurements. The self‐discharge characteristics were monitored at open circuit starting at time *t*_oc_. e,f) Upon self‐discharge, the voltage drop versus the square root of time, normalized by the different starting potentials ranging from 1.4 to 2 V in steps of 0.2 V.

For the cyclic voltammetry in **Figure** **4**a, the supercapacitors exhibited similar oxidation current, but the reduction current was lower in the device with aprotic electrolyte than the one with protic electrolyte. This result using the PANI–rGO cathode was in agreement with earlier analyses in Figure 2 on PANI without rGO. Irreversible oxidation to the oligo‐aniline occurred in aprotic electrolyte, and the subsequent current did not fully recover to the initial level, even when rGO was added to mitigate fragment disconnections in the active materials.

The disparity between the oxidation and reduction currents in the aprotic electrolyte was evident in the galvanostatic charge–discharge (GCD) measurement, as shown in Figure 4b and S5, Supporting Information. The supercapacitor with the aprotic electrolyte showed a CE of less than 66.7%, where the discharge output lost a third of the input charge. In contrast, the device with the protic electrolyte showed a CE of 99.0%, showcasing nearly identical charging and discharging capacities. It reached a gravimetric capacity of 180 mAh g^−1^, compared to the 162 mAh g^−1^ observed in the aprotic electrolyte. This 180 mAh g^−1^ capacity of this PANI–rGO cathode represented 61% of the theoretical capacity (294 mAh g^−1^) for neat PANI. If we exclude the mass of rGO and account for PANI contribution alone, the cathode measured at a current density of 6.5 mA cm^−2^ showed a capacity of 257 mAh g^−1^, reaching 87% of the maximum theoretical capacity. When the current density was decreased to 3.3 mA cm^−2^, the capacity was raised further to 280 mAh g^−1^ (95% of the theoretical capacity), although the CE under this condition was reduced to 94%.

One drawback of the protic electrolyte was the increased equivalent series resistance (ESR) in Figure 4c. The ESR was 25 Ω for the protic device and 10 Ω for the aprotic one, due to the reduced conductivity of the electrolyte with protic additive (Figure 3a and temperature dependence in Figure S6, Supporting Information). The increased ESR would adversely affect the maximum power that the device could deliver. Nevertheless, despite this trade‐off in power, the protic additive enhanced the device's energy density and stability in terms of suppressing self‐discharge and extending cycle life.

Self‐discharge refers to the gradual loss of stored charges overtime and can be caused by charge redistribution, Ohmic leakage, and Faradaic side reactions.^[^ ^27^, ^28^, ^29^ ^]^ The impacts of charge redistribution and Ohmic leakage can be minimized by holding the cells at a fixed potential for a brief duration. In our measurement scheme illustrated in Figure 4d, the devices were charged to the target voltage and held at that voltage for 3 min, and then the drop in open‐circuit potential was monitored. The target voltages were varied from 1.4 to 2 V, and the potential decay was normalized to the initial voltage value in Figure 4e,f for devices with protic and aprotic electrolytes, respectively. The potential decay overtime *t* was fitted to the equation^[^ ^27^ ^]^ *V*(*t*) = *V*_*0*_ − *m* $\sqrt{t}$, where *V*_0_ is the initial voltage and *m* is the rate of voltage drop due to Faradaic side reactions. Higher *m* rates were observed with increasing applied voltage, attributed to accelerated side reactions. For the device with the protic electrolyte, the extracted *m* values were in the range of 1.3–3.3 mV s^−0.5^. In contrast, with the aprotic electrolyte, the rate of potential decay was shown to change around the 60 s^0.5^ time point, with an initial rapid potential drop of 5.5–51 mV s^−0.5^, which then transitioned to a slower decay rate of 0.9–2.3 mV s^−0.5^. The two regimes suggested there was fast degradation in the beginning phase, likely due to the fragmentation of PS state in the aprotic environment. After this phase, the decay rates slowed down and fell within the same range as in the protic electrolyte. The self‐discharge characteristics confirmed that the protic electrolyte was crucial to suppress side reactions.

**Figure 5:**
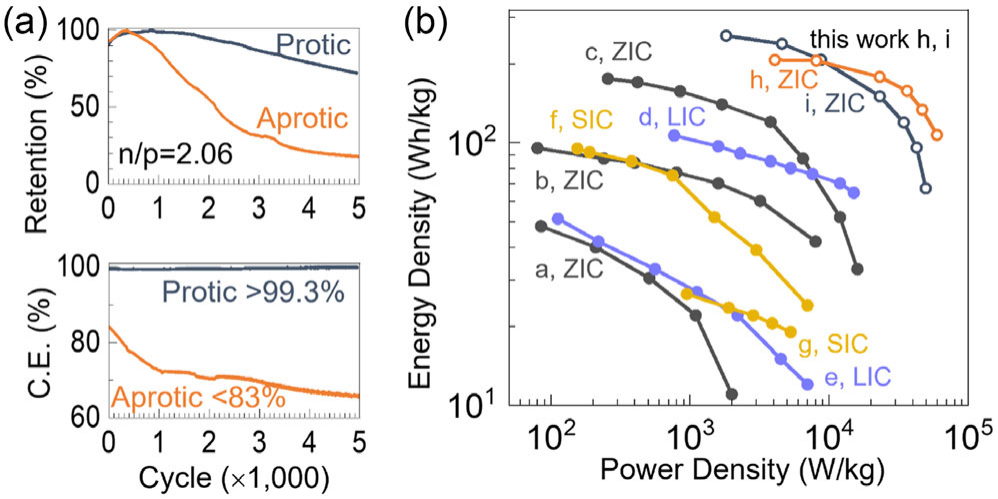
Comparison of PANI–rGO||Zn supercapacitors with protic or aprotic electrolyte. a) Retention of discharging capacity and b) Coulombic efficiency versus redox cycles. Charge–discharge cycles were carried out at a current density of 13 mA cm^−2^. (b) Gravimetric energy and power densities of metal ion capacitors. ZIC: Zn‐ion capacitor; LIC: Li‐ion capacitor; and SIC: Na‐ion supercapacitor. The device labels correspond to Table 1.

**Figure** **5**a compares the capacity retention and CE as a function of redox cycles between 0 and 2 V. With the protic electrolyte, the supercapacitor retained 80% of its original capacity after 3,850 cycles, whereas the device with the aprotic electrolyte fell below 80% after 1,200 cycles. The CE of the aprotic device was less than 83% and further decreased with more redox cycles, while the protic device showed CE over 99.3% throughout 5000 cycles (data for CE extraction shown in Figure S7, Supporting Information). The cumulative capacity of the protic supercapacitor was measured to be as 474 mAh cm^−2^, by summing up the discharge capacity of all redox cycles before the retention metric fell below 90%. This cumulative capacity was better by 6.6‐ and 13‐fold compared to previous reports on lithium and sodium ion capacitors using EDL carbon, respectively (as shown in Table S1, Supporting Information).^[^ ^30^, ^31^, ^32^ ^]^

In Figure 5b and **Table** **1**, the gravimetric energy and power densities achieved in this work are compared to other metal ion capacitors using PANI cathodes as reported in recent literature. The prior state‐of‐the‐art devices displayed a maximum energy density under 180 Wh kg^−1^ at a power density under 1 kW kg^−1^. In our devices, expanding the voltage window to 2 V in nonaqueous electrolytes improved the maximum energy density to 255 Wh kg^−1^ at a power output of 1.8 kW kg^−1^ and the maximum power density to 50 kW kg^−1^ with energy density at 100 Wh kg^−1^. High conductivity through rGO in the PANI composite contributed to the enhanced power density.

**Table 1:** Compositions and metrics of metal ion capacitors with PANI cathode.

Serial no.a)	Electrode (cathode||anode)	Electrolyte	Potential [V]	Power (W kg^−1^) at max energy	Max energy [Wh kg^−1^]	References
1	EG‐PANI || Zn	2M ZnSO_4_(aq)	0.1–1.5	85	48	[33]
2	NSG/PANI || Zn	1M ZnSO_4_(aq)	0–1.6	80	95.4	[34]
3	RAP || Zn	1M Zn SO_4_(aq)	0.2–1.9	256.8	175.5	[35]
4	PANI || CNF	1M LiPF_6_ in EC/DEC/DMC	2–4	769	106.5	[36]
5	TALP || graphite	1M LiPF_6_ in EC/DEC/DMC	0–1.6	112.3	51.3	[37]
6	PANI || PTCD	1 m NaClO_4_ in EC/DMC	0–3	155	95	[38]
7	PANI–NaMP || AC	2M H_2_SO_4_(aq)	0–1.8	950	26.5	[39]
8	PANI–rGO || Zn	0.5 m Zn(CF_3_SO_3_)_2_ in ACN	0–2	4,000	206	This work
9		0.5 m Zn(CF_3_SO_3_)_2_ in ACN with PG (95:5)	0–2	1,800	255	

a) EG: electrochemically exfoliated graphene. NSG: nitrogen and sulfur co‐doped graphene. RAP: reduced graphene oxide anchored with 1‐anthraquinonesulfonic acid sodium‐salt‐decorated PANI. CNF: carbon nanofiber. TALP: tungsten‐anion‐linked polyaniline. PTCD: perylene‐3,4,9,10‐tetracarboxylic acid dianhydride. NaMP: NaMnPO_4_. AC: activated carbon. rGO: reduced graphene oxide. EC: ethylene carbonate. DEC: diethyl carbonate. DMC: dimethyl carbonate. ACN: acetonitrile. PG: propylene glycol. Aq: aqueous water solvent.

When comparing our devices with and without the protic additive, the aprotic electrolyte delivered more energy in the high‐power region, because of its superior ionic conductivity. Conversely for power outputs below 9 kW kg^−1^, the protic electrolyte was not limited by kinetics and facilitated better energy densities than its aprotic counterpart. Taking all aspects of energy/power densities, cycle life, and self‐discharge together, the combination of PANI–rGO and protic electrolyte offered a straightforward approach to implement state‐of‐the‐art cathodes in metal‐ion supercapacitors.

## 3 Conclusion

This study demonstrated the importance of protic moieties in enabling reversible redox reactions through the utilization of fully oxidized PANI in cathodes. In situ Raman spectra revealed the structural changes associated with different oxidation pathways that the protic‐stabilized PS form of PANI was highly stable during redox cycling, but the fragmented PS was not reversible, lowering the discharge capacity. Therefore, the role of protic additive was to screen the PANI by hydrogen bonds from side reactions. This understanding laid the foundation for the design principle of a PANI composite cathode in a protic electrolyte, and the resulting devices achieved the best‐in‐class performance for zinc‐ion supercapacitors. The enhanced capacity, cycle life, and self‐discharge characteristics hold promise for expanding the use of PANI as high‐capacity cathodes in energy‐storage cells. Given that PANI is well known and readily available, this work in understanding and improving its redox stability will be widely useful for various applications in supercapacitors, batteries, and other electrochemical devices including sensors and actuators.

## 4 Experimental Section

4.1

4.1.1

##### Synthesis of Emeraldine Salt PANI

A solution of 252 mL of chloroform (SK Chemicals, 99%) and 346 mL of deionized water was blended in a double jacket reactor. Subsequently, 33.4 mL of hydrochloric acid (SAMCHUN, 35%) and 38 mL (0.41 mol) of aniline were added dropwise to the reactor and the temperature was reduced to −5 °C. Another mixture solution, 0.55 m of ammonium persulfate (JUNSEI, 95%) and 1.2 m of hydrochloric acid in deionized water, was added dropwise to the reactor using a dropping funnel at −5 °C for 2 h, followed by stirring for 30 min. The product was washed and filtrated with deionized water and methanol (SK Chemicals, 99%) and dried in a vacuum oven at 60 °C, which yielded 15.7 g of ES PANI. Finally, the obtained product was purified through Soxhlet extraction with ACN (SAMCHUN, 99.5%) for 12 h to remove the oligomers and the low molecular weight fraction of polymer, to achieve conductivity of 39 S cm^−1^.

##### Characterization of Emeraldine Salt PANI

^1^H nuclear magnetic resonance (NMR) spectrum was obtained using a Jeol JNM‐ECZ600r NMR spectrometer (Figure S8, Supporting Information), and UV–vis absorption spectra was measured using a Jasco V‐770 UV–vis spectrometer to confirm the structure of the synthesized PANI. Electrical conductivities were characterized by a Nittoseiko Analytech Loresta‐GX MCP‐T700 resistivity meter.

##### Preparation of Electrolytes

The nonaqueous electrolyte was 0.5 m of zinc trifluoromethanesulfonate, also known as zinc triflate (Zn(OTf)_2_, Sigma‐Aldrich, 98%), dissolved in ACN (Fisher Scientific, 99.9%) at room temperature. The protic additive was PG (Fisher Scientific) mixed into the electrolyte solvent at 19:1 ACN:PG volume ratio.

##### Fabrication of Zinc‐Ion Supercapacitors

Two types of cathodes were fabricated, one for the samples examined in Raman spectroscopy, and the other was further optimized to increase the electrode capacity. In the Raman samples, PANI was mixed with carbon black (CB) and polyvinylidene fluoride (Solvay PVDF 5130) in a weight ratio of 8:1:1. For all other samples, the PANI, rGO (MTI Corporation), and polyvinylidene fluoride were mixed at a weight ratio of 7:3:1. Both powder mixtures were suspended in 1‐methyl‐2‐pyrrolidinone (Fisher Scientific, >99.5%) to make a solution of 12.5% solids by weight. The solution was deposited on a carbon cloth (AvCarb,1071 HCB) and annealed at 100 °C for 6 h, to reach a solid loading around 1 mg. Before cell assembly, another round of annealing was carried out at 230 °C for 1 h.

For the anode, the Raman samples used an as‐bought zinc foil (Leishent, thickness of 10 μm). For all other samples, to increase zinc utilization and thereby the energy density of the devices, zinc was deposited on a graphite sheet by electroplating under 12.5 mA cm^−2^ for 6 min. A 0.5 m zinc triflate electrolyte was used as the electroplating solution, and the deposited zinc film was ≈5 μm thick.

The anode and cathode were separated by a glass microfiber separator (Cytiva, Whatman GF/A). After adding 160 μL of the electrolyte, the sandwich structure was encapsulated in coin cells (CR2032) by a hydraulic crimping machine (MTI corporation, MSK‐110).

##### Statistical Analysis

The specific details of the cells are listed in **Table** **2**. Each measurement was carried out at least five times in each condition.

**Table 2:** Dimensions and materials loading of the devices measured in this work.

	Components	Dimensions	Notes
Figure 2	• Cathode loading of PANI:CB:PVDF (8:1:1): 1.0 mg in protic electrolyte 1.1 mg in aprotic electrolyte	Round pieces with diameters of 1.4 cm for both cathode and anode	The electrolyte was 0.5M Zn(OTf)_2_ in acetonitrile with or without 5% of propylene glycol.
	• Anode: Zn foil (10 μm)		
Figure 3	• Cathode loading (various PANI:rGO ratio, while all had 9% PVDF): 1.2 mg (10:0); 0.6 mg (9:1) 1.3 mg (8:2); 1.6 mg (7:3) 1.0 mg (5:5)		
	• Anode: Zn foil (10 μm)		
	• 160 μL electrolyte		
Figure 4	• Cathode loading of PANI:rGO:PVDF (7:3:1): 0.9 mg in protic electrolyte 1.4 mg in aprotic electrolyte		
	• Anode loading of Zn: 2.41 mg		
	• 160 μL electrolyte		
Figure 5a	• Cathode loading of PANI:rGO:PVDF (7:3:1): 3.2 mg in protic electrolyte 3.0 mg in aprotic electrolyte		
	• Anode loading of Zn: 2.41 mg		
	• 160 μL electrolyte		
Figure 5b	• Cathode loading of PANI:rGO:PVDF (7:3:1): 0.9 mg in protic electrolyte 1.4 mg in aprotic electrolyte		Calculations of gravimetric energy/power densities accounted for the loading of PANI–rGO, but excluded the weights of current collectors, binder, and separator.
	• Anode loading of Zn: 2.41 mg		
	• 160 μL electrolyte		
	• Separator: 18.7 mg		
	• Carbon cloth current collectors: 19.7 mg		

##### Raman Spectroscopy and Electrochemical Characterization

The Raman spectroscopy was measured via a Renishaw inVia microscope. The excitation light source was a helium–neon 633 nm laser outputting a power of 1.7 mW with a spot size of 1.5 μm. Raw spectra were processed by smoothing over 10 points after baseline subtraction.

Electrochemical measurements were conducted by a potentiostat (SP‐200 Bio‐logic). Cyclic voltammetry was scanned over the potential range of 0–2 V at a scan rate of 5–90 mV s^−1^. GCD measurements were conducted under constant current densities in the range of 6.5–65 mA cm^−2^. Electrochemical impedance spectroscopy was performed in the frequency range from 50 mHz to 200 kHz to obtain the ESR and ion conductivity. Cycle lifetime was measured using a Neware battery tester at the current density of 13 mA cm^−2^.

Based on cyclic voltammetry data, the specific capacitance was calculated by $C=\frac{1}{w_{\text{Cathode}}\text{ Δ}V\nu }\int_{V_{1}}^{V_{2}}IdV$, where $w_{\text{Cathode}}$ is the loading of the cathode, *V*_1_ and *V*_2_ are the starting and ending potentials in the discharge portion of the measurement, *I* is the current at each potential, Δ*V = V*_2_ *–V*_1_ is the potential window, and *ν* is the voltage scan rate. Gravimetric capacity of the cathode was extracted using the equation of Capacity_cathode_ =I×tdwCathode, where $t_{d}$ is the discharge time at each current density. The cumulative capacity was calculated through the summation of discharge capacity for which the CE was over 99% and the discharge capacity retention was over 90% at the current density of 13 mA cm^−2^.

The *n*/*p* ratio was calculated through dividing the capacity of the zinc anode by the discharge capacity of the cathode. The capacity of the zinc anode was calculated by multiplying the measured weight of the electrodeposited zinc with the theoretical gravimetric capacity of zinc: Capacity_Anode_ =820mAh g−1×wanodeA, where $w_{\text{anode}}$ is the anode Zn loading, and *A* is the area of the anode.

The energy density was calculated from GCD characteristics using the equation of *E* = (∫VIdt)/$w_{\text{Cathode}}$, where *V* is the potential, and *I* is the applied current. The power density was calculated based on the equation of *P* = *E*/*t*_d_, where *t*_d_ is the discharge time.

## Conflict of Interest

The authors declare no conflict of interest.

## Author Contributions

**Chanho Shin**: Conceptualization (equal); Formal analysis (lead); Investigation (lead); Writing—original draft (lead). **Eun Hye Lee**: Investigation (equal); Methodology (equal); Writing—review & editing (supporting). **Hyeong Ju Eun**: Methodology (supporting). **Jinwook Jung**: Investigation (supporting). **Jong H. Kim**: Supervision (equal); Writing—review & editing (supporting). **Tse Nga Ng**: Conceptualization (lead); Formal analysis (supporting); Funding acquisition (lead); Supervision (lead); Writing—review & editing (lead). **Chanho Shin** and **Eun Hye Lee** contributed equally to this work.

## Supporting information

Supplementary Material

## Data Availability

The data that support the findings of this study are available from the corresponding author upon reasonable request.
