# Supplementary material for: Protic Stabilization Engenders High Energy Density and Long Cycle Life in Polyaniline–Zinc Supercapacitors
Source: Small Sci. 2024 Sep 1;4(11):2400295. doi: 10.1002/smsc.202400295 (PMC11935264; doi:10.1002/smsc.202400295)
Supplement: Supplementary file 1 — Supplementary Material [file SMSC-4-2400295-s001.pdf]

Protic Stabilization Engenders High Energy Density and Long Cycle Life in Polyaniline-Zinc Supercapacitors

Chanho Shin[1,2+], Eun Hye Lee[3+], Hyeong Ju Eun[3], Jinwook Jung[1], Jong H. Kim[3#], Tse Nga Ng[1,2*]

[1] Program in Material Science and Engineering, University of California San Diego, 9500 Gilman Drive, La Jolla, California 92093, United States.

[2] Department of Electrical and Computer Engineering, University of California San Diego, 9500 Gilman Drive, La Jolla, CA 92093, United States.

[3] Department of Molecular Science and Technology, Ajou University, Suwon 16499, Republic of Korea.

[+] C.S. and E.L. contributed equally to this work.

*Email: tnn046@ucsd.edu; [#]Email: jonghkim@ajou.ac.kr

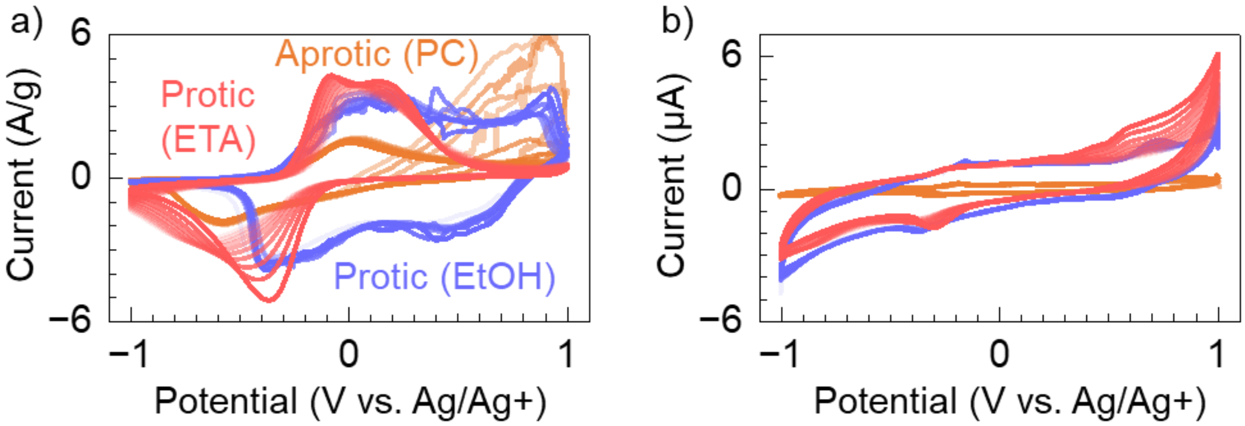

Figure S1. Current-voltage characteristics with aprotic and protic electrolytes versus Ag/Ag^{+} reference. For the aprotic electrolytes, 0.1 M of Zn triflate was dissolved in propylene carbonate (PC) solution. For the protic electrolytes, 0.5 M Zn triflate was dissolved in mixed solution which composed of 95% acetonitrile and 5% ethanol (EtOH) or ethanolamine (ETA). (a) PANI cathode and Zn anode, and (b) Glassy carbon electrodes.

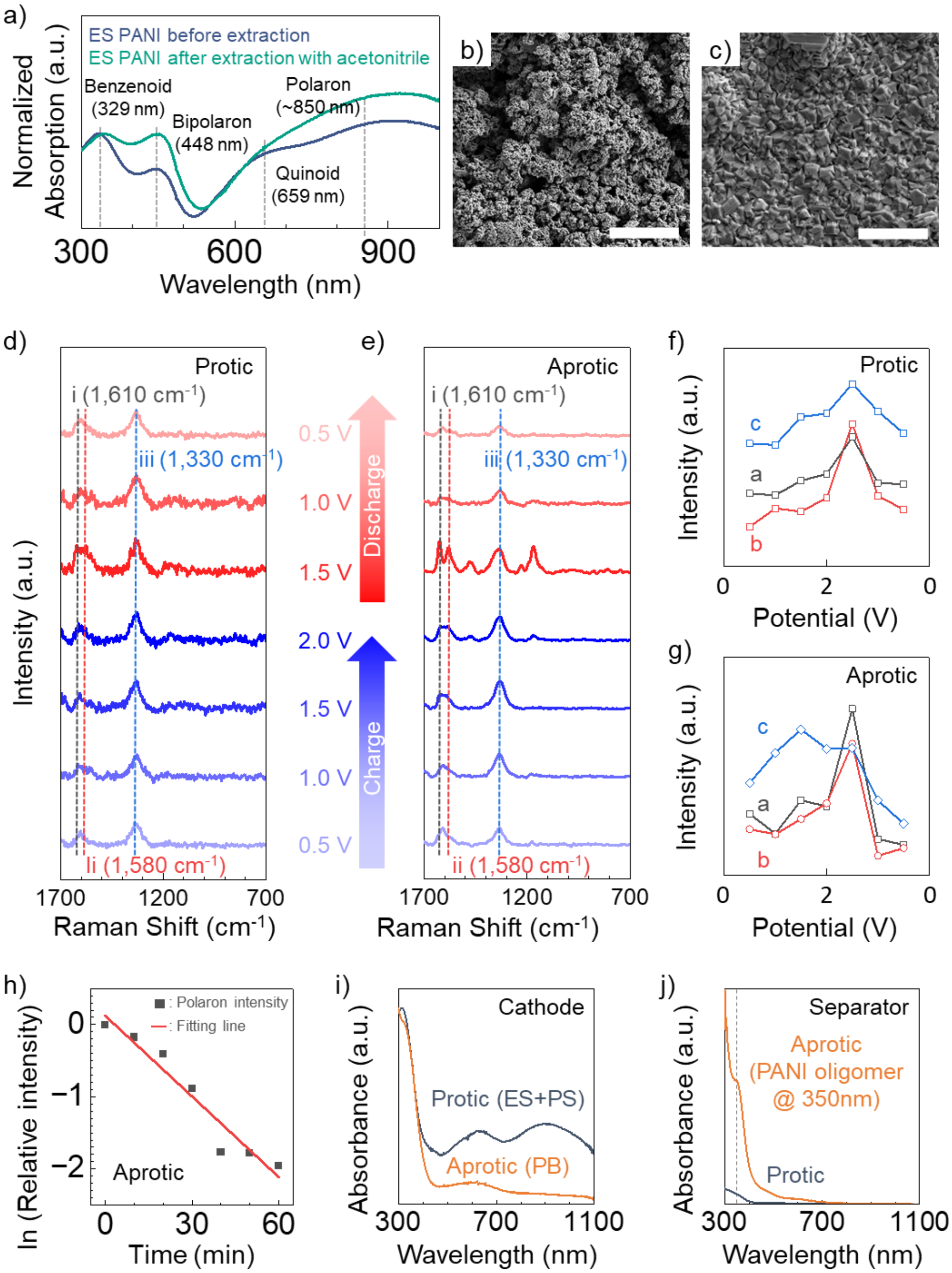

Figure S2. Characterization of polyaniline. (a) Ultraviolet-visible (UV-Vis) spectra of ES PANI in dimethyl sulfoxide (1 mg/ml) after soxhlet extraction with acetonitrile. (b) Scanning electron microscopy images of (b) polyaniline coated on carbon cloth and (c) electroplated zinc on graphite. Scale bars: 20 μm. In-situ Raman spectra data during cyclic voltammetry measurement at a scan rate of 5 mV/s in the potential range of 0-2 V with (d) protic and (e) aprotic electrolyte. (f, g) Based on the Raman spectra, the peak

intensities for benzenoid (1,610 cm^{-1}), quinoid (1,580 cm^{-1}), and polaron (1,330 cm^{-1}) were plotted to analyze the changes in molecular structures. (h) Time dependence of polaron intensity at an applied potential 2 V for 1 hour in aprotic electrolyte. The fitted line was calculated according to the equation of $I=I_0$ exp(-bt). UV-Vis spectra was measured on (i) the cathode and (j) the separator after applying 2 V for 1 hour. While ES and PS forms of PANI were detected on cathode in protic electrolyte, only the PB form of PANI was detected on cathode in aprotic electrolyte. Additionally, the separator collected from the device with the protic electrolyte did not show discernible absorption peak. However, the separator collected from the device with aprotic electrolyte showed PANI oligomer absorption peak at 350 nm.

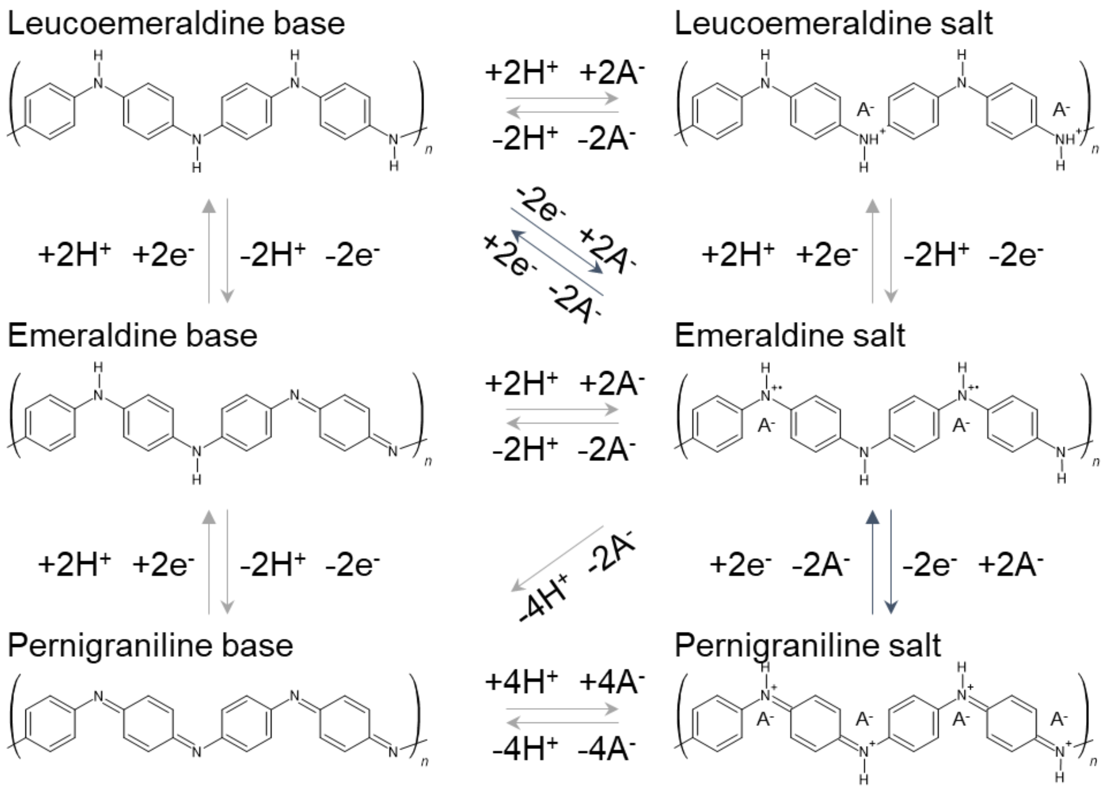

Figure S3. Various states of polyaniline during oxidation and protonation.

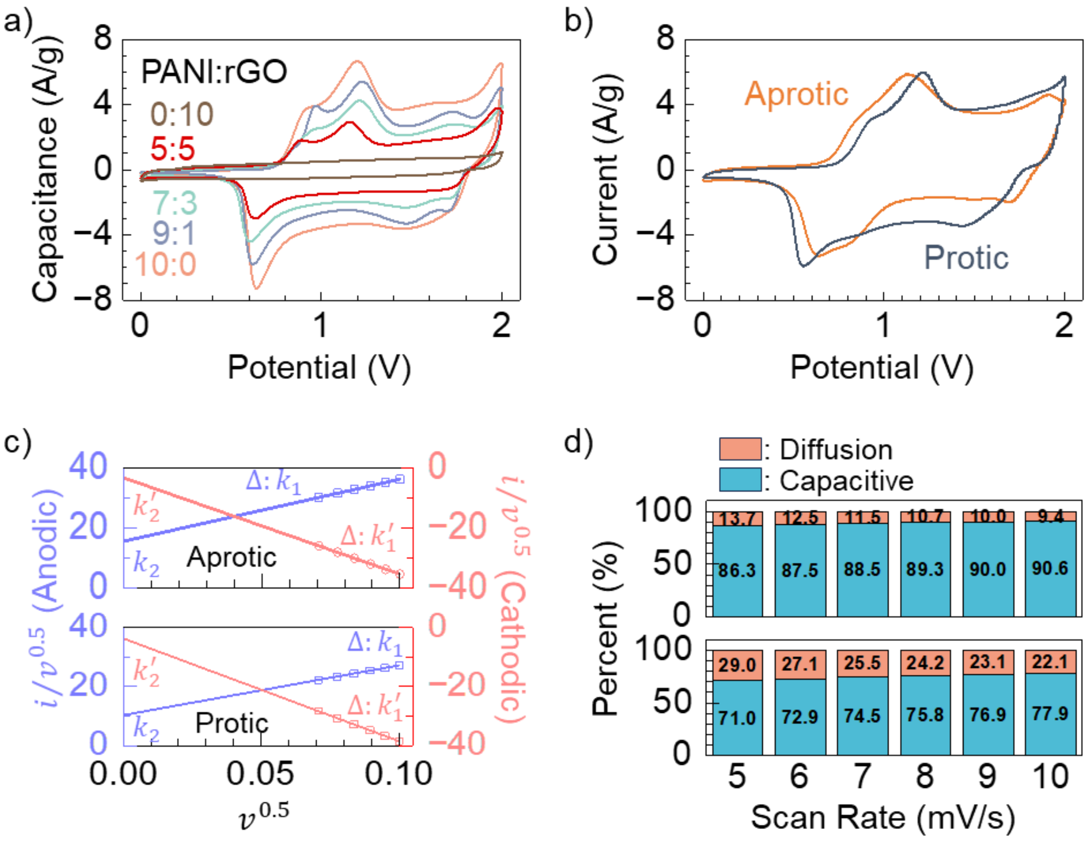

Figure S4. (a) The cyclic voltammetry data was measured with the several ratios of cathode materials consisting of ES polyaniline and reduced graphene oxide at a scan rate of 10 mV/s. The cyclic voltammetry data of mixture cathode (ES PANI:rGo = 7:3) was measured at scan rate of 5 to 10 mV/s to analyze the contribution of capacitive and diffusion controlled current. (b) Each curve shows the cyclic voltammetry data with (black line) and without (orange line) protic additive at scan rate of 10 mV/s. (c) The capacitive and diffusion-controlled contributions to the cyclic voltammetry data were derived by k_1 and k_2 from the Dunn model equation of '$i(\nu)=k_1\nu + k_2\nu^{0.5}$' (*Science* **2014**, vol. 343, p.1210). The k_1 and k_2 are the slope and the y-intercept in $i(V)/v^{0.5}$ versus $v^{0.5}$ plot. (d) The ratios of diffusion and capacitive-controlled current at each scan rate.

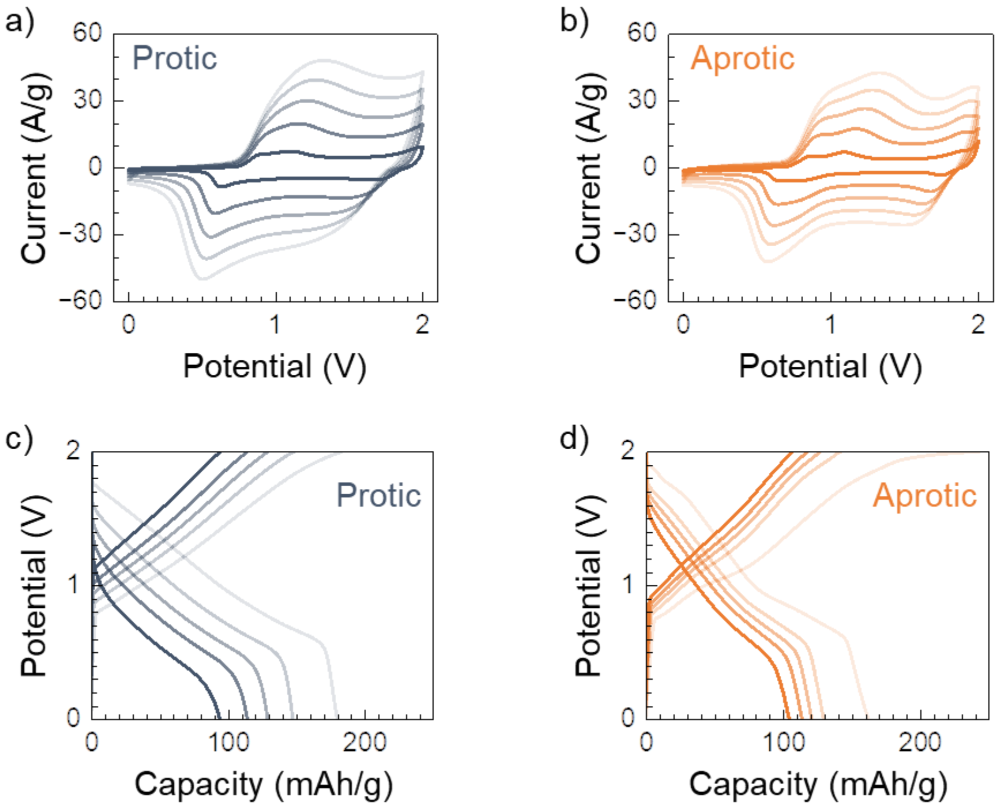

Figure S5. Performance comparison of zinc ion capacitor with different electrolytes. Cyclic voltammetry was measured at scan rates of 10-90 mV/s (a) with and (b) without propylene glycol. Galvanostatic charge-discharge measurements were taken at current densities of 6.5-65 mA/cm^2 (c) with and (d) without propylene glycol.

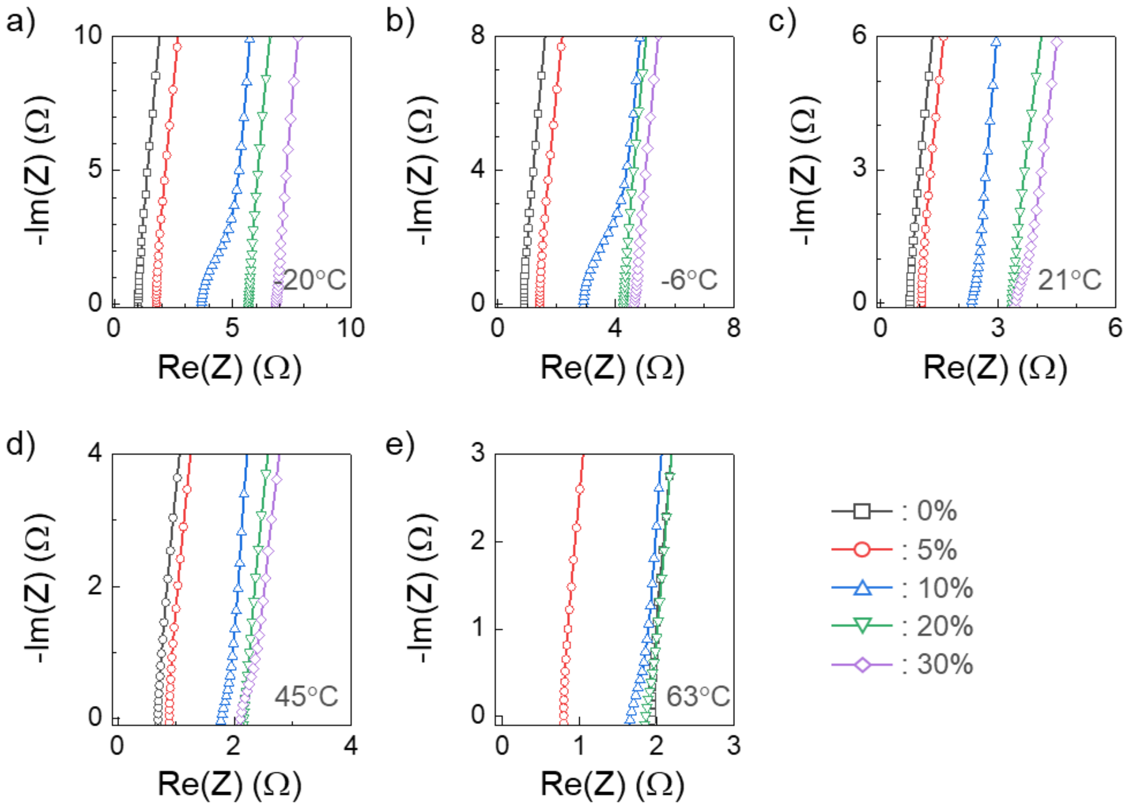

Figure S6. Nyquist plot of 0.5M zinc triflate film with different propylene glycol volume ratios. Each Nyquist data was measured at (a) -20°C, (b) -6°C, (c) 21°C, (d) 45°C, and (e) 63°C in the frequency range of 100 mHz to 100 kHz.

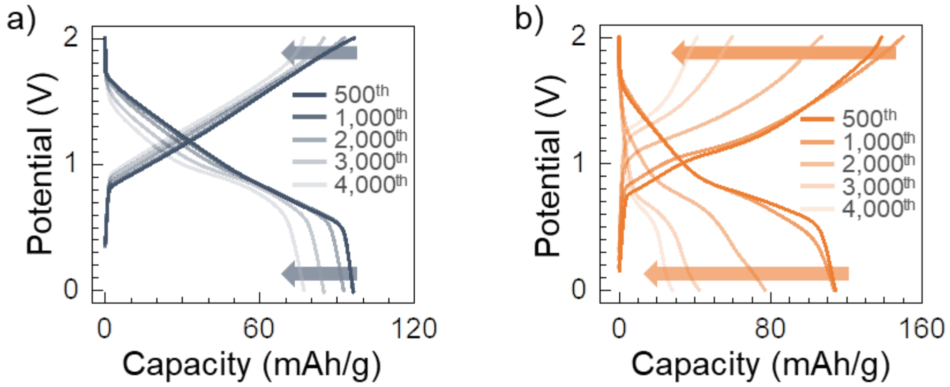

Figure S7. The change of galvanostatic charge-discharge curves during cycling at current density of 13 mA/cm^2: (a) Protic electrolyte, (b) Aprotic electrolyte.

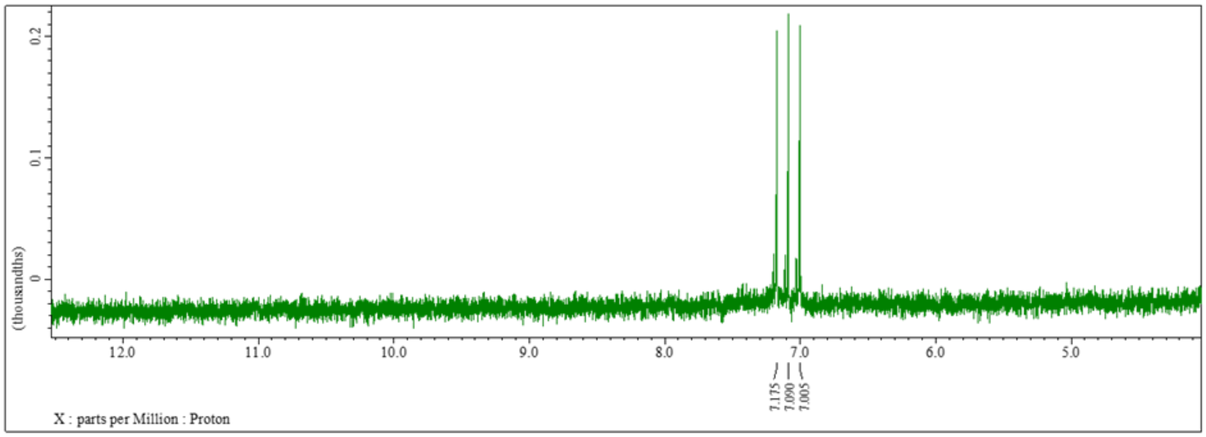

Figure S8. ^{1}H NMR spectrum of the synthesized polyaniline using dimethyl sulfoxide-d_6 (δ 7.090 (t, $^{1}J_{NH}$ = 51.1 Hz)).

Table S1. Structure and metrics of metal ion capacitors on cycling stability and cumulative capacity.

Electrode (Anode||Cathode)	Electrolyte	Current Density (mA/cm^2)	Areal Capacity (mAh/cm^2)	Cycle Lifetime (Cycles)	Cumulative Capacity (mAh/cm^2)	Ref. # in text
Fe3O4/G||Graphene	$LiPF_6$ in EC/DEC/DMC	4.7	0.50	180	72	31
Na-C||AC	$NaClO_4$ in EC/DEC	15	0.31	124	37	32
Zn||PANI-rGO	0.5M $Zn(CF_3SO_3)_2$ in ACN with PG (95:5)	13	0.18	2700	474	30

※ The cumulative capacity was calculated from the sum of GCD cycles for which the Coulombic efficiency was over 99%, and the discharge capacity retention exceeded 90%.
